# miRkwood: a tool for the reliable identification of microRNAs in plant genomes

**DOI:** 10.1186/s12864-019-5913-9

**Published:** 2019-06-28

**Authors:** Isabelle Guigon, Sylvain Legrand, Jean-Frédéric Berthelot, Sébastien Bini, Delphine Lanselle, Mohcen Benmounah, Hélène Touzet

**Affiliations:** 1 University of Lille - bilille, F-59000 Lille, France; 2 University of Lille, CNRS, UMR 8198 - Evo-Eco-Paleo, F-59000 Lille, France; 3 University of Lille, CNRS, INRIA, UMR 9189 - CRIStAL, F-59000 Lille, France

**Keywords:** Micro-RNAs, Small RNA-seq, Plant genome, AGO-IP

## Abstract

**Background:**

MicroRNAs (miRNAs) play crucial roles in post-transcriptional regulation of eukaryotic gene expression and are involved in many aspects of plant development. Although several prediction tools are available for metazoan genomes, the number of tools dedicated to plants is relatively limited.

**Results:**

Here, we present miRkwood, a user-friendly tool for the identification of miRNAs in plant genomes using small RNA sequencing data. Deep-sequencing data of Argonaute associated small RNAs showed that miRkwood is able to identify a large diversity of plant miRNAs and limits false positive predictions. Moreover, it outperforms current tools such as ShortStack and contrary to ShortStack, miRkwood provides a quality score allowing users to rank miRNA predictions.

**Conclusion:**

miRkwood is a very efficient tool for the annotation of miRNAs in plant genomes. It is available as a web server, as a standalone version, as a docker image and as a Galaxy tool: http://bioinfo.cristal.univ-lille.fr/mirkwood

**Electronic supplementary material:**

The online version of this article (10.1186/s12864-019-5913-9) contains supplementary material, which is available to authorized users.

## Background

Since their discovery in animals and plants, microRNAs (miRNAs) have been shown to play pivotal roles in growth and development of organisms. Canonical miRNAs are endogenous ~ 21 nt small RNAs that regulate key developmental processes or the response to environmental stresses at the post-transcriptional level by mediating the cleavage of the target messenger RNAs (mRNAs) and/or by inhibiting their translation. So far, about 300 miRNAs have been annotated in the model plant *Arabidopsis thaliana*. The most conserved ones play crucial roles in plant patterning, cell identity and development by coordinating the expression of transcriptions factors and F-box proteins. The most recently evolved miRNAs target mRNAs encoding a broader range of proteins and are involved in diverse processes, including plant response to environmental cues (see for review [1, 2]).

Many aspects of the biogenesis and evolution of miRNAs differ between animals and plants. For example, unlike animal miRNAs, which are mainly found in introns or exons from coding genes, most plant miRNAs are encoded by discrete genes. Moreover, miRNAs are released from their precursors (pre-miRNA) using distinct pathways in the two kingdoms. Animals use a nuclear Drosha RNAse III enzyme to liberate pre-miRNA from primary transcript. After nuclear export of pre-miRNAs, they are cleaved into miRNA/miRNA* duplexes (miRNA* being the passenger strand) by a cytoplasmic Dicer enzyme. In plants, both steps of cleaving are performed by a single nuclear Dicer-like protein (DCL). *MIR* genes (i.e. genes producing miRNAs) give rise predominantly to canonical 21-nt miRNAs, which are generated by DCL1 from hairpin precursors. Beside 21 nt miRNAs, as shown in *A. thaliana* and rice, DCL3 is capable of generating 24-nt long miRNAs (lmiRNAs) from pre-miRNAs, which are loaded into AGO4 proteins and direct cytosine methylation to trigger transcriptional gene silencing [3–5]. Also, miRNA precursors are more heterogeneous in plants than in animals, varying greatly in size and structure [6]. These differences have justified dedicated approaches for miRNA gene finding.

High-throughput sequencing of cDNA-libraries derived from endogenous small RNAs (sRNA-seq), is a widely used and powerful method for the discovery and annotation of miRNA-producing genes. In particular, sRNA-seq has uncovered many young and non-conserved miRNAs (of both 21 and 24 nt) that are less expressed than their conserved counterparts and therefore more difficult to detect. However 24-nt lmiRNAs could be easily confounded with heterochromatic small interfering RNAs (hc-siRNAs) that are very abundant in plant sRNA-seq libraries. Indeed, both are 24 nt in length, DCL3 dependent, loaded into AGO4, and direct cytosine DNA methylation. Nevertheless, these two types of sRNAs present important differences, notably with respect to their origins. Like canonical miRNAs, lmiRNAs are produced from hairpin pre-miRNAs, while hc-siRNAs are produced from dsRNAs originating from transposable element (TE) loci and DNA repeats. Moreover, hc-siRNAs specify DNA methylation in *cis* at the same or very similar locus from which they originate, while lmiRNAs can direct DNA methylation at target loci that are different from those that produce them, typically in trans [5].

Although many computational tools dealing with sRNA-seq data in animals are available, the number of tools calibrated for plants is relatively limited (e.g. [7–10], see [11] for review). Moreover, none of these tools is available as a web-server or offer a GUI. Considering this gap, we have developed miRkwood that is specifically designed for plant miRNAs. It is able to face the diversity of plant pre-miRNAs (producing canonical and lmiRNAs) and it is optimised to take advantage of their distinctive properties [12–14]. Moreover, miRkwood provides information related to multi-mapping miRNAs. miRkwood is available as a web-server offering an intuitive and comprehensive user interface to navigate the data, as well as many export options to conduct further analyses on a local computer. Moreover, it is available in command line with a Docker container and as a Galaxy tool.

## Implementation

### Workflow overview

**Fig. 1:**
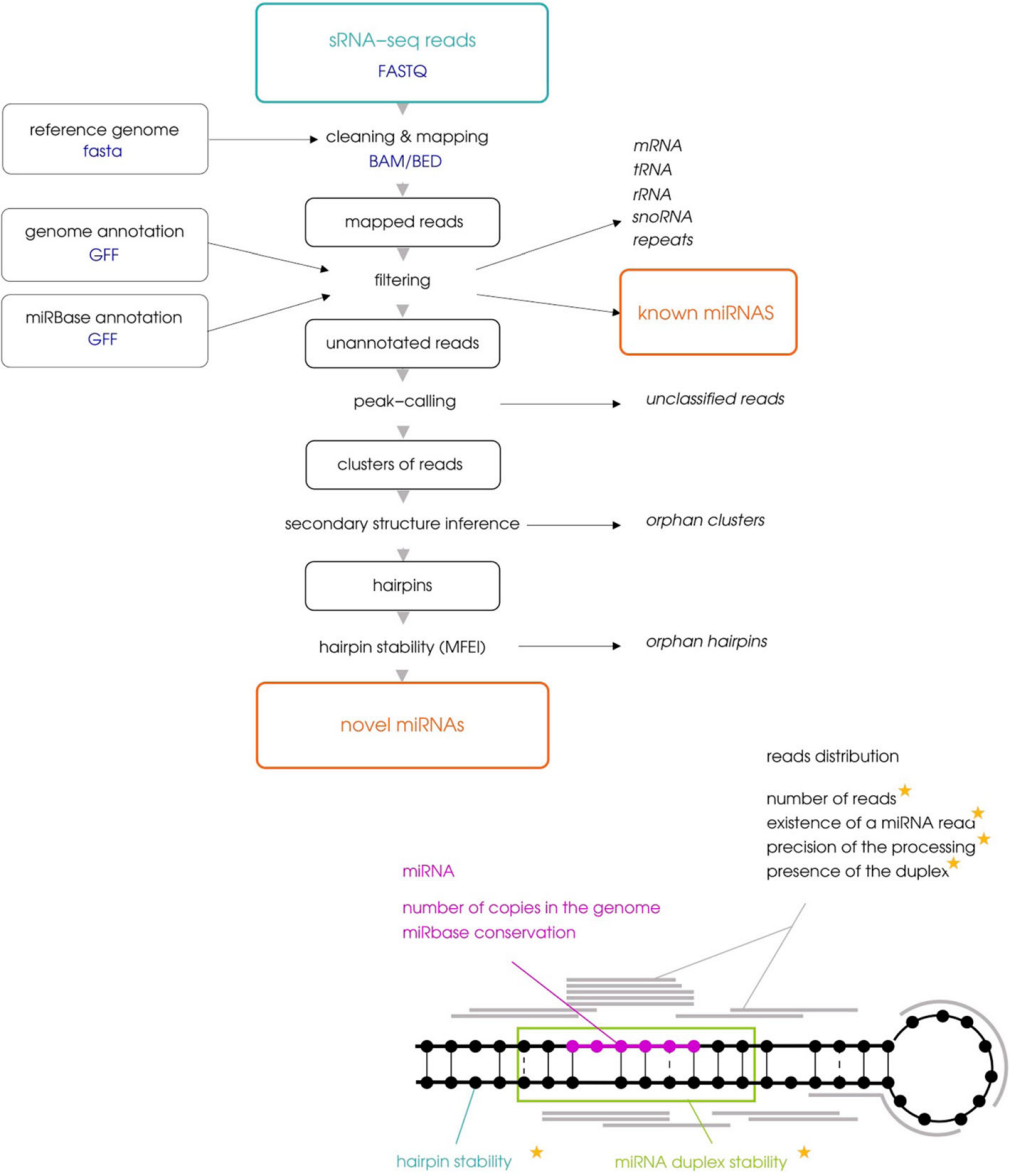
Overview of miRkwood methodology. The workflow describes the main steps covered by miRkwood, such as detailed in the Implementation section. The six criteria defining the quality score ranging from 0 to 6 (expressed in stars) as well as the conservation of the miRNA with miRBase are represented on the hairpin

miRkwood implements a complete workflow that combines information of both read positions and secondary structures of miRNA precursors (Fig. 1).

#### Alignment and filtering

The starting point is to map small RNA sequencing reads on the reference genome to produce an alignment file. This can be done by the user with any standard short read mapper, such as Bowtie2 [15] or BWA [16] for example. When annotation of the reference genome is available, it is advised to filter out the alignments based on the existing annotation provided in GFF files. This allows to discard false positive predictions due to degradation products, and speed up the computation. We offer to mask coding regions, tRNAs, rRNAs and snoRNAs using their genomic coordinates (see Additional file 1, Section 1). We also propose to remove reads that are mapped to more than 5 loci (customizable threshold) on the reference sequence. This allows to avoid spurious predictions due to TEs. All these options are activated by default, but remain optional.

#### Identification of known miRNAs

When available, genome coordinates of miRNA precursor sequences such as provided in miRBase [17] for example, may be used to detect known miRNAs that are expressed in the sequencing data.

#### Peak-calling

The next step of miRkwood is to locate expression signals into the set of reads that have not been eliminated in the previous step. For that, we have developed a method that is both scalable and takes advantage of the secondary structure of the precursors. First, we identify areas in the genome that have been enriched in aligned reads using a classical k-mean clustering method. Then, for each such peak we look for RNA duplexes by testing whether the sequence can bind to a sequence located in the vicinity (up to 350 nt upstream or downstream of the peak) by establishing C-G, A-U and G-U interactions. This condition ensures that the peak can be part of a potential stem-loop structure, and reduces the number of peaks to consider in the next step. This search is achieved using a custom program (see Additional file 1, Section 2).

#### Secondary structure of the hairpin precursor

After peak detection, miRkwood aims at determining which sequences can fold into a stable stem-loop structure. This step is critical because precursor stem-loops are very heterogeneous in plants. The main idea is to use the RNALfold software of the RNA Vienna package [18] to identify locally stable secondary structures without any prior knowledge. This computation is time consuming, which is why we have made sure that only a limited number of peaks are kept from the previous step. We then select stable secondary structures that are compatible with a miRNA stem-loop in terms of length and number of base pairings. In Section 3 of Additional file 1, we provide an advanced discussion on the parameters used and the accuracy of the approach.

#### Thermodynamic stability of the hairpin precursor

For each hairpin structure found, we compute the MFE (minimal free energy), AMFE (adjusted MFE) and MFEI (MFE index), which are all interrelated. The MFE is calculated using the Matthews-Turner nearest neighbor model implemented in RNAeval [18]. miRkwood offers the option to select only sequences with MFEI < − 0.6. Indeed, more than 96% of miRBase precursors have an MFEI smaller than − 0.6, whereas pseudo-hairpins show significantly larger values of MFEI in general (see Additional file 1, Section 4). The significance of the stability of the sequence can also be determined by comparison with random sequences with the same dinucleotide composition [19].

At this point, the selected loci form the set of *candidate miRNA precursors.* The remaining of the workflow is devoted to the computation of additional criteria that bring further evidence to evaluate quality of the candidate miRNA precursors. For that, we have introduced a total of six criteria, that are gathered into a quality score ranging from 0 to 6 (expressed in stars). Candidates with a score of 6 fulfill all criteria and are considered highly reliable.

The first criterion is the stability of the secondary structure of the precursor. The five other criteria relate to the distribution of mapped reads along the locus. They allow to determine if this distribution presents a typical 2-peaks profile corresponding to the guide miRNA and the passenger miRNA respectively: number of reads, existence of the miRNA, precision of the precursor processing, presence of the miRNA:miRNA* duplex, stability of the the miRNA:miRNA* duplex.

#### Criterion 1: stability of the hairpin precursor

This criterion is met when the MFEI of the structure is smaller than − 0.8. This threshold covers 83% of miRBase pre-miRNAs, whereas it is observed in less than 13% of pseudo hairpins (see Additional file 1, Section 4, Table 2).

#### Criterion 2: number of reads

This criterion is met when the locus has either at least 10 reads mapping to each arm, or at least 100 reads mapping in total. This criterion is inspired from miRBase definition for high-confidence miRNAs (http://www.mirbase.org/blog/).

#### Criterion 3: existence of the miRNA

The most common read is selected as the guide miRNA sequence if its frequency is at least 33%. Otherwise, we do not define any mature miRNA sequence for the locus.

When the miRNA is properly defined, we consider the three following properties.

#### Criterion 4: precision of the precursor processing

At least 75% of reads start in a window [− 3,+ 3] centered around the start position of the miRNA, or [− 5,+ 5] around the pairing position on the opposite arm of the stem-loop. These parameters are conform to observations in miRBase.

#### Criterion 5: presence of the miRNA:miRNA* duplex

There is at least one read in the window [− 5,+ 5] around the pairing position on the strand of the passenger miRNA.

#### Criterion 6: stability of the duplex

In Kurihara and Watanabe, 2004 [20] it is reported that the guide miRNA and the passenger miRNA form a duplex with two nucleotide overhangs, and base-pairing between the miRNA and the other arm is extensive. We formalize it with the usage of miRdup [21], that assesses the stability of the miRNA:miRNA* duplex by machine learning (with random forests). It was trained on miRBase Viridiplantae V20 with default parameters.

With this score system, hairpin precursors with no clear miRNA locus have a score of at most 2. Hairpin precursors with a guide miRNA and no passenger miRNA have a score of at most 5. Reaching a score of 6 means that the locus shows the expression of both the guide miRNA and the passenger miRNA, and that its secondary structure (hairpin and duplex) is consistent with this expression.

Lastly, miRkwood provides two additional pieces of information for each prediction. First, it checks if the miRNA (when it exists) could possibly originate from a duplication. For that, it reports whether the sequence is present elsewhere in the genome, and whether this alternative location corresponds to another miRNA precursor found by miRkwood. Second, it checks whether the miRNA is conserved through evolution by comparison with a database of known miRNAs (see Additional file 1, Section 5). Alignments are performed with the piccolo alignment tool, which is particularly effective for processing short sequences, such as miRNAs [22].

### Output

**Fig. 2:**
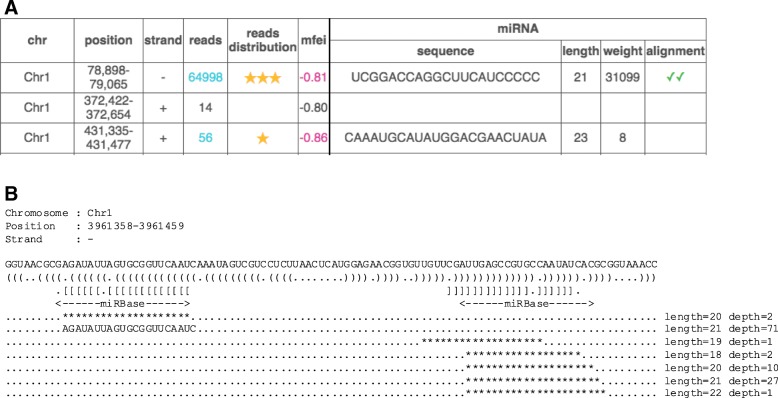
Examples of outputs. **a**: Summary table. The first six columns are for the stem-loop precursor. Reads is the number of reads that are mapped to the locus (when this number fulfils criterion 2, it is highlighted in turquoise), reads distribution is the total score achieved by criteria 4–6, mfei is the MFEI of the secondary structure of the precursor (when its value is smaller than − 0.8, it is highlighted in pink, in accordance with criterion 1). The four last columns are for the miRNA. Sequence is the sequence of the guide miRNA, when its exists (criterion 3), and length is its length. Weight is the number of reads corresponding to the miRNA normalized by the total number of occurrences of the sequence in the genome. Alignment indicates if there is an alignment with mature plant miRNAs in miRBase. This cell is checked when such an alignment is found, and doubled checked if it overlaps the miRNA locus in the precursor. **b**: Read cloud. The read cloud is the visual representation of a candidate miRNA precursor. The first line is the sequence of the precursor. The second line is the stem-loop structure in bracket-dot format. The third line with square brackets and dots highlights the miRNA duplex (when the miRNA is well-defined). The fourth line indicates the position of the alignments with the miRBase sequence, when found. In the remaining lines, each ********** string is a unique read. Its length and its depth (its number of occurrences in the set of reads) are reported at the end of the dotted line. The mature miRNA, when one is defined, is written in full letters. In this example, the locus has a total of 114 reads, corresponding to 7 unique reads. The most frequent read is AGAUAUUAGUGCGGUUCAAUC, with 71 copies (representing 62% of reads). Following criteria 3, it is selected as the guide miRNA. Its length is 21 nucleotides. The passenger miRNA is UUGAGCCGUGCCAAUAUCACG, supported by 27 reads. As expected, its position is shifted by two nucleotides compared to the guide miRNA. Lastly, we found two distinct alignments with miRbase, whose positions coincide to the guide and the passenger miRNAs respectively, which is a good indicator that the miRNA duplex is evolutionary conserved

Results are presented as an overview web page where miRNA precursors are displayed in a table, with each row corresponding to a pre-miRNA, and each column to a specific feature (Fig. 2a). By default, results are sorted by position. It is possible to have them sorted by quality. One can visualise the full report for a pre-miRNA prediction by clicking on the name of the sequence. This report sums up all previously computed information including, but not limited to, the 2D secondary structure of the pre-miRNA, the components of the quality score, the alignment of short reads on the pre-miRNA (reads cloud) and all alignments with miRBase sequences, when they exist (Fig. 2b).

### Export

Search results are available in a variety of formats: CSV (supported by all spreadsheets, such as Excel), FASTA, dot-bracket notation (FASTA sequence plus the secondary structure), GFF, text report in ORG-mode and read clouds.

### Availability

miRkwood is implemented in Perl and C. It offers an intuitive and comprehensive user interface to navigate the data. For now, the web version has 12 reference genomes: *Arabidopsis lyrata, Arabidopsis thaliana, Brassica napus, Brassica rapa, Glycine max, Lotus japonicus, Medicago truncatula, Oryza sativa, Populus trichocarpa, Solanum lycopersicum, Sorghum bicolor and Vitis vinifera*. Most of them are supplemented by two GFF files, that are used to filter out the reads in the alignment filtering step (see above). The first one contains the genome coordinates of annotated CDS, tRNAs, rRNAs and snoRNAs, and is used to apply masking options. The other GFF file compiles all miRNAs and precursors of miRNAs available in miRBase V21. User’s reads should be previously mapped on one of those 12 assemblies, and the coordinates of the resulting alignments stored in a custom BED file. A tool to automatically convert SAM/BAM file to the BED file is provided with miRkwood. The database for the alignments to known miRNAs is miRBase V21 (file mature.fa), but can be updated as new versions of miRBase are released.

The stand-alone version is freely distributed under the GNU Affero General Public Licence (v3.0). The code is available on GitHub: https://github.com/miRkwood-RNA/miRkwood. The user can provide any genome and any annotation file of his/her choice. To make the installation and the usage easier, we offer a Galaxy wrapper (available on the Test Toolshed) and a Docker image.

Finally, besides the sRNA-seq mode, miRkwood also offers an ab initio mode, which allows the prediction of miRNA precursors from assembled expressed sequences or short genomic sequences (up to 100 kilobases on the website).

## Methods

### Datasets

To evaluate the performance of miRkwood and benchmark against ShortStack [10], a dataset composed of 6.2 million Illumina raw reads and obtained from inflorescences from *A. thaliana* previously published by Wang et al. (2011) [23] was downloaded from the SRA-NCBI database (accession number SRX058635). AGO1 and AGO4 associated small RNAs (AGO1-IP and AGO4- IP) data obtained from the same biological sample were downloaded from the GEO database (accession numbers GSM707682 and GSM707690 for AGO1-IP and GSM707686 for AGO4-IP). They were composed of 969,586, 884,610 and 2,171,046 non-redundant reads, respectively.

### Pre-processing of reads and miRNA predictions

Adaptors were removed from the sequencing reads using Cutadapt (version 1.8.3) [24] and reads were cleaned using Prinseq (version 0.20.4) [25] with specified parameters: -min_len 18 -max length 30 -min_qual_mean 30. Quality of the cleaned Illumina reads was checked using FastQC (version 0.11.4) [26]. Prior to miRkwood analysis, reads were mapped on the *A. thaliana* genome (TAIR10, [27]) using Bowtie (version 1.1.2) [15] with specified parameters: -v 0 --all --best --strata, and sam output files were converted to bed format using the dedicated tool, mirkwood-bam2bed.pl provided with miRkwood. For comparison, miRNAs were also predicted using ShortStack (version 3.8.2) [10] with default parameters.

### Analysis of predictions

Pre-miRNA predictions were compared to pre-miRNAs annotated in the *A. thaliana* genome assembly using the “intersect” function from the Bedtools suite (version 2.27.1) [28]. Predicted pre-miRNAs were considered as annotated, i.e. corresponding to a known pre-miRNA in the *A. thaliana* genome, when the overlap was larger than 30 bp. Pre-miRNAs predicted by ShortStack were then compared to the whole set of *Viridiplantae* mature miRNAs deposited into miRBase using Exonerate (version 2.4.7) [29], allowing at most 3 mismatches/indels and no consecutive indels. It was not necessary to perform this analysis for pre-miRNAs predicted by miRkwood since it is already included into the pipeline.

A miRNA prediction was considered as associated with AGO1 or AGO4 when the miRNA sequence was observed at least once in the dataset (without mismatch nor additional nucleotide). When the mean number of reads in the two AGO1-IP datasets was greater than that observed in the AGO4-IP dataset the miRNA were considered as preferentially associated to AGO1, and vice versa. For this calculation, the number of reads was normalized by the number of unique reads in each of the datasets (reads per million).

The colocalization between pre-miRNA predictions and TE annotations in the *A. thaliana* genome was evaluated using the “intersect” function from the Bedtools suite (with an overlap of at least 1 bp) and the recent deep annotation of TEs in *A. thaliana* [30]. Predictions obtained by miRkwood and ShortStack were considered as identical when pre-miRNAs presented an overlap of at least 30 bp (also defined using the “intersect” function from the Bedtools suite).

## Results and discussion

We evaluated the performance of miRkwood using a sRNA-seq dataset from *A. thaliana* previously published (see methods) [23]. Following the cleaning step, we conserved 4.8 million reads (i.e. 77% of the raw reads), representing 2.6 million non-redundant reads. MiRkwood predicted a total of 2049 pre-miRNAs/miRNAs, including 87, 41, 42 and 70 with a quality score equal to 6, 5, 4 and 3, respectively. MiRkwood allowed to retrieve 173 miRNA precursors from *A. thaliana*. Since we performed miRNA predictions from only one sRNA-seq experiment representing a single tissue (inflorescence) at a single time point, we did not expect to find all the 326 miRNAs annotated in the *A. thaliana* genome (miRBase, V22). Predictions below a quality score of 3 will not be further considered, since they do not systematically exhibit a clearly identifiable miRNA sequence.

To validate the predictions, the association between the predicted miRNAs and AGO1 was evaluated using deep-sequencing data of AGO1 associated small RNAs (AGO1-IP), AGO1 being the main argonaute protein used in the miRNA pathway loading canonical 21 nt miRNAs. We also evaluated the association of the predicted miRNAs with AGO4 that binds to lmiRNAs of 24 nt. The performance of miRkwood was compared to that of ShortStack [10], one of the most popular and effective tools for the prediction of miRNAs in plants, particularly because of its ability to avoid false positive predictions, compared to other tools [9, 14].

### Detection of high confidence miRNAs with high sensitivity

**Fig. 3:**
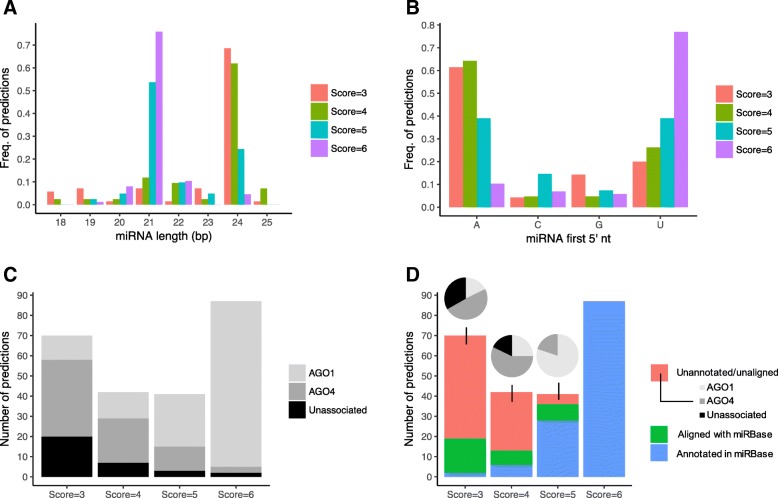
Features of miRNA predictions obtained by miRkwood. **a**: Length of predicted miRNAs. **b**: 5′ nt of the predicted miRNA sequence. **c**. AGO association of the different predictions according to their score. **d**. Annotation of the predicted miRNAs and alignment to miRNA sequences from miRBase. Association of the unannotated and unaligned sequences with AGO proteins are shown in pie charts

MiRNA predictions with a quality score of 6 were mainly 21 nt long (76%) (Fig. 3a), initiated predominantly with a 5′ U (77%, Fig. 3b) and were mainly associated with AGO1 (94%, Fig. 3c). Similar results, although less pronounced, were observed for miRNAs predictions with a score equal to 5: 54% were 21 nt long, 39% started with a 5′ U and 63% were associated with AGO1. Hence, high score predictions seem to predominantly correspond to canonical 21-nt miRNAs produced by DCL1 and associated with AGO1.

Although not all of the 87 miRkwood predictions reaching a quality score of 6 were associated with AGO1, we observed that they were all annotated in the *A. thaliana* genome (including 3 predictions preferentially associated with AGO4), suggesting that they are real miRNAs (Fig. 3d). In contrast, only 28 of the 41 predictions with a quality score of 5 were annotated in the reference genome. However, among the 13 unannotated predictions, 8 presented homology with plant miRNAs deposited in miRBase and the 5 remaining miRNAs were all validated using the AGO1-IP data (including one 24 nt prediction preferentially associated with AGO4) suggesting that they are indeed bona fide miRNAs.

**Fig. 4:**
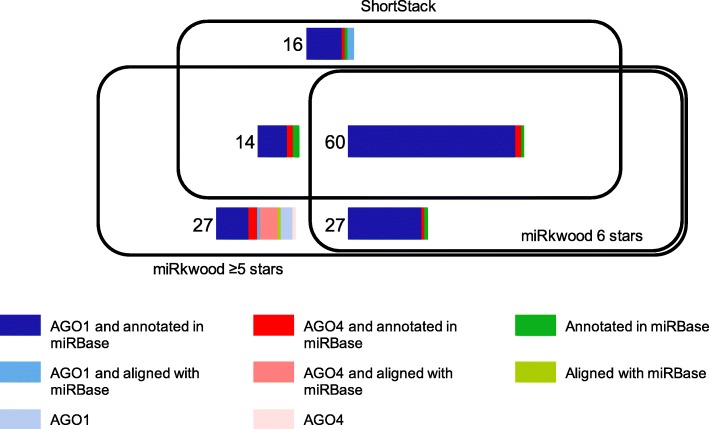
Comparison of the predictions obtained by miRkwood and ShortStack. The Venn diagram of the predictions obtained by both tools with two different score thresholds for the miRkwood results (≥5 stars and equal to 6 stars). Within the rectangles, the bar plots describe the predictions: blue and red are for miRNA predictions associated with AGO1 and AGO4, respectively. Green is for predictions not-associated with AGO1 or AGO4 proteins. The dark and intermediate shades correspond to miRNA predictions which were annotated in miRBase or could be aligned with miRBase sequences, respectively. The light shade indicates predictions which were neither annotated nor aligned with miRBase

From the same dataset, ShortStack provided 90 predictions (Fig. 4), i.e. 29.7% less predictions than miRkwood set up with a quality score ≥ 5. All of them were annotated in the reference genome and/or aligned with known miRNAs from miRBase and/or associated with AGO proteins and could be consequently considered as bona fide miRNAs. Two-thirds (60) of the predictions from miRkwood set up with a quality score threshold equal to 6 were obtained by both tools and one-third of the predictions were specific to each one (27 and 30 for miRkwood and ShortStack, respectively, Fig. 4). These results show that at this threshold, miRkwood produces comparable results as ShortStack in terms of numbers of predicted miRNAs. When the quality score threshold was set up to ≥5, the number of specific miRNAs predicted by miRkwood increased to 54, whereas that of ShortStack sharply decreased (16). At this threshold, in addition to a substantial number of specific predictions, miRkwood is able to detect the major part (82%) of the ShortStack predictions.

In brief, when miRkwood is set up with a quality score ≥ 5, both tools mainly detect canonical miRNAs and limit the number of false positives, but miRkwood is able to predict substantially more miRNAs than ShortStack (128 vs. 90).

### Identification of novel miRNAs

Decreasing the quality score threshold in miRkwood presented a negative impact on the predictions, since it increased the proportions of unassociated predictions with AGO proteins (and so unvalidated). Indeed, 17 and 29% of unassociated predictions were observed for predictions with a 4 and 3 score, respectively, vs. 2, 7%, for predictions of a quality of 6 and 5, respectively. Nonetheless, we observed that miRNAs predictions with a score of 4 and 3 were predominantly 24 nt long (62 and 69%, respectively, Fig. 3a), started mainly with a 5′ A (64 and 61%, respectively, Fig. 3b), and were mainly associated with AGO4 (52 and 54%, respectively, Fig. 3c), which corresponds to features of 24-nt lnmiRNA or hc-siRNAs loaded into AGO4 [31].

Among the 42 predictions with a quality score of 4, only 13 were annotated or presented similarities with known miRNAs, leaving 29 putative novel predictions. Among them, 23 were associated with AGO proteins (7 with AGO1 and 16 with AGO4). Among the 70 predictions with a score of 3, only 19 were annotated or aligned with known miRNAs, and 34 of the 51 remaining miRNAs were associated with AGO proteins (9 with AGO1 and 25 with AGO4). Thus, even at the threshold scores of 3 and 4, miRkwood provides predictions that may correspond to bona fide miRNA. Beside, a large part of the predictions presents sRNA sequences associated with AGO4, implying they could correspond to lmiRNAs or to hc-siRNAs. Considering that hc-siRNAs emerge from TE loci, we tested the intersection between these predictions and the deep TE annotations in the *A. thaliana* genome [30]. We observed that among the 42 predictions with a score of 3 or 4 that are not annotated in the reference genome, not aligned with known plant miRNAs, and not associated with AGO1, but which are associated with AGO4, only 20 colocalized with TEs, suggesting that a significant part of the AGO4 associated predictions could be lmiRNAs.

Concerning the comparison of the results with ShortStack, even if decreasing the threshold score to 4 or 3 did not deeply reduce the number of specific predictions to ShortStack, it increased the number of those specific to miRkwood (95 and 165, respectively, data not shown).

In summary, decreasing the threshold score to 4 or 3 may increase the proportion of false positives, but potentially offers in return the detection of novel canonical miRNAs and lmiRNAs.

## Conclusions

We took advantage of the distinctive properties of plant pre-miRNAs/miRNAs to develop miRkwood, a dedicated tool for miRNA gene discovery in plant genomes. The performance analysis showed that miRkwood is able to predict miRNA with high sensitivity and can be tuned either to limit the number of false positives or to broadly identify novel miRNAs. Contrary to ShortStack, it provides a quality score for each prediction (ranging from 0 to 6) allowing users to rank miRNA predictions. miRkwood is available either as a web server, with a user-friendly interface, as a standalone version, as a docker image or as a Galaxy tool. We believe that miRkwood will be a useful tool for biologists interested in the identification of miRNAs in plant genomes.

## Availability and requirements

Project name: miRkwood

Project home page: http://bioinfo.cristal.univ-lille.fr/mirkwood, https://github.com/miRkwood-RNA/miRkwood

Contact: mirkwood@univ-lille.fr

Operating system(s): Unix or web server

Programming languages: Perl, C and C++

Other requirements for the Unix version: bedtools (v2.14.2 or higher), Vienna package (v2.1.6-1), Blast+ (2.2.25+ or higher), miRdup (1.2 or higher), VARNA (v3-91 or higher, optional).

License: GNU Affero GPL

Other requirements for the web version: none. The version of miRBAse will be regularly updated. New plant genomes can be added upon request.

Any restrictions to use by non-academics: none

## Additional file

Additional file 1: Detailed description of some of the miRkwood steps. (PDF 166 kb)

## Data Availability

Not applicable.

## Abbreviations

AGO-IP: Immunoprecipitation of argonaute proteins
hc-siRNAs: Heterochromatic small interfering RNAs
lmiRNAs: Long micro-RNA
miRNA: Micro-RNA
pre-miRNA: Precursor of micro-RNA
sRNA-seq: Small RNA sequencing
TE: Transposable element
