# Supplementary material for: miRkwood: a tool for the reliable identification of microRNAs in plant genomes
Source: BMC Genomics. 2019 Jun 28;20:532. doi: 10.1186/s12864-019-5913-9 (PMC6599362; doi:10.1186/s12864-019-5913-9)
Supplement: Supplementary file 1 — Detailed description of some of the miRkwood steps. (PDF 166 kb) [file 12864_2019_5913_MOESM1_ESM.pdf]

Additional file 1: Detailed description of some of the miRkwood steps

1 Alignment filtering

When annotation of the reference genome is available (in a GFF file), we offer the possibility to discard reads whose alignment overlaps an annotated element. For that, we use the BEDtools intersect function and remove each read that overlaps a feature by at least 1 nt. Selected features are “CDS” for coding regions, “tRNA” for transfer RNA, “rRNA” for ribosomal RNA, “snoRNA” for small nucleolar RNA.

As for the “known miRNAs”, we use genome coordinates of miRBase precursor sequences and we select any read that is entirely included in a precursor (miRNA_primary_transcript in miRBAse file).

2 Peak calling

To locate expression signals into the set of reads, we have developed a method that is both scalable and takes advantage of the secondary structure of the precursors.

- First, we scan the set of reads to detect regions with high read coverage (twice the average).
- Second, we identify statistically significant peaks within these regions using K-means approach.
- Finally, for each peak we test if the sequence can bind to a neighboring sequence, which is a necessary condition to belong to a hairpin. The goal is to eliminate peaks that are not likely to occur in a hairpin loop. This step is performed by dynamic programming, with a method inspired from Smith and Waterman algorithm for local alignment [1]. We define a local score that reflects the binding affinity between two regions. For each pair of nucleotides, we assign a weight: 1 for G↔U, 2 for A↔U, 3 for G↔C is 3, and -2 for all other pairs (mismatches or matches). Watson-Crick pairs and wobble pairs have a positive weight defined as the number of hydrogen bonds involved in the pairing, whereas all other pairs have a negative weight. Insertions or deletions of a nucleotide have a penalty of -3. The algorithm then searches for the best affinity between the 21 nt region surrounding the peak summit and the flanking region (400 nucleotides).

3 Secondary structure of the hairpin precursor

3.1 Algorithm

The first problem to address when searching for microRNAs precursors is the typology of their

secondary structures. Plant pre-miRNAs are known to be more variable in size and structural features than those of animals. To illustrate this, Figure 1 shows the size distribution of all plant pre-miRNAs (*Viridiplantae,* 6150 sequences) extracted from miRBase (Release 20) [2]. The size ranges from 43 to 938 nt, the mean is 146 nt and the standard deviation 76.

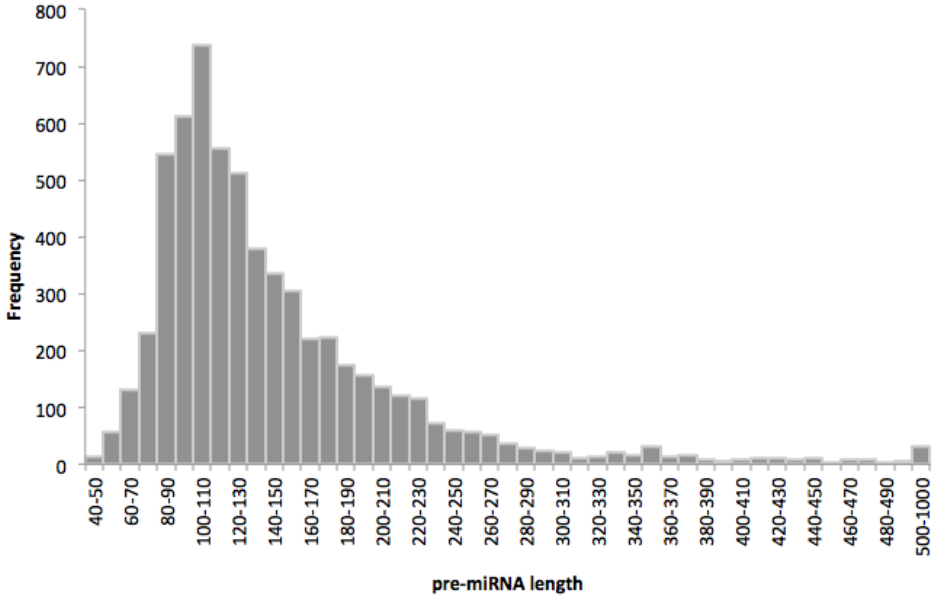

Figure 1: Frequency distribution of the length of plant pre-miRNAs (miRBase, V20)

As for the secondary structures, we investigated the optimal folding structure with minimum free energy of each plant pre-miRNA sequence. For that, we computed the MFE structure with RNAfold [3], and parsed these structures to examine their shape. We found out that for 1604 of them (>25%) the MFE structure is not a pure stem-loop. However, these structures do contain a stem-loop, and the terminal loop for some local structural elements. Figure 2 shows such an example.

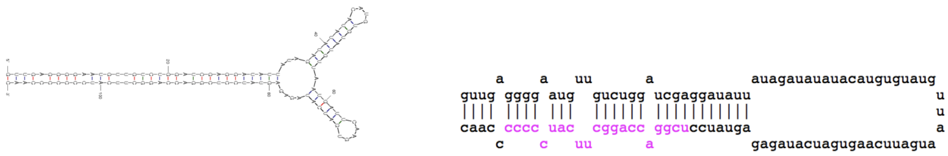

Figure 2 : Optimal secondary structure of ath-mir165a, computed with RNAfold (left), and the stemloop secondary structure provided in miRBase (right)

Considering these two points, we have developed a three-step strategy to identify putative miRNA precursors :

- scan the peak sequence for locally stable secondary structures with RNALfold [4]. We used a sliding window of length 400 (option –L). This value has been chosen to cover more than 98% of pre-miRNAs present in miRBase. RNALfold is guaranteed to return only optimal MFE structures and does not require any other parameters, such as the maximal size of a bulge.
- filter out the output of RNALfold and select all secondary structures that might contain a stem-loop. This is done with a custom program, that uses three parameters :
    - the minimum length of the pre-miRNA, set to 70
    - the maximum length of the pre-miRNA, set to 400
    - the size *s* of a sliding window and a number *n* of base pairs, such that the hairpin should contain at least one window of length *s* with at least *n* base pairs. We set *s*=20 and *n*= 17, which corresponds to the fact that the duplex formed by the mature miRNA and the star miRNA is significantly stable.
- group overlapping predictions (more than 50% positions in common), and select the best candidate according to this rule: if a prediction is included into another prediction, select the longest one whose MFEI is smaller than -0.8. In all other cases, select the prediction with lower MFEI (see definition of the MFEI below). The other structures are kept as alternative foldings for the same candidate.

3.2 Evaluation on pseudo-hairpins

To evaluate the accuracy of our algorithm and see whether it is able to distinguish true precursors from pseudo-hairpins, we show some experimental results on positive and negative datasets.

The two positive datasets are composed of known plant pre-miRNAs extracted from miRBase V20. We have selected two subsets: *Plant pre-miRNAs* and *High confidence plant pre-miRNAs.*

Plant pre-miRNAs: All pre-miRNAs from the clade *viridiplantae* without any ambiguous character, such as N, R, K,… : This gives 6,070 sequences (out of a total amount of 6,150 plant sequences in miRBase).

High confidence plant pre-miRNAs : This is a subset of the preceding dataset, which corresponds to miRBase entries based on deep sequencing data (http://www.mirbase.org/blog/2014/03/high-confidence-micrornas). We have selected all plant sequences without any ambiguous character: This gives 561 sequences (out of 566

sequences).

As for the negative samples, we have used two pseudo-hairpins datasets: *PlantMiRNAPred pseudo-hairpins* and *Triplet-SVM pseudo-hairpins*, that have both been previously defined to train miRNA classifiers.

PlantMiRNAPred pseudo-hairpins: This dataset comes the plant pseudo hairpin dataset used in plantmirnapred [5], and was downloaded from http://nclab.hit.edu.cn/PlantMiRNAPred. This is a set of 2,122 sequences extracted from protein coding sequences of *Arabidopsis thaliana* and *Glycine max.* This collection of pseudo-hairpins is also used in [6] and [7]. Since a threshold of 70 nt is used on the size of the stem-loop and that some sequences are longer than the stem-loop structure, we selected all sequences whose length is larger than or equal to 80 nt to avoid spurious true negatives. This gives a total amount of 2,062 sequences.

Triplet-SVM pseudo-hairpins: This dataset comes from the training set used by Triplet-SVM [8] and MiPred [9], and was downloaded from http://bioinfo.au.tsinghua.edu.cn/software/mirnasvm/Triplet-svm-predictor.htm. This is a set of pseudo-hairpins constructed on human messenger RNA sequences. We selected all sequences whose hairpin length is greater than or equal to 70 nt. This gives a total amount of 3,381 sequences.

We tested the performance of our approach to find precursors sequences on each of these data sets. Results are displayed in Table 1. They show that our approach finds a stem-loop structure for more than 85% of pre-miRNAs present in miRBase. False negatives come mainly from short stem-loops (less than 70 nt) and more importantly incomplete sequences in miRBase. Not surprisingly, results are even better with high confidence miRBase precursors: it reaches 98%.

The selectivity can be evaluated on the two pseudo-hairpins datasets, plantMiRNApred and triplet-SVM. We observe less than 21% false positive predictions. So, we are able to discard more than 75% of pseudo-hairpins.

	Total number of sequences in the dataset	Number of sequences with at least one hairpin structure found
**Positive datasets**		
plant mirbase	6070	5215 (85.9%)
high confidence plant mirbase	561	550 (98.0%)
**Negative datasets**		
plantMiRNApred	2062	418 (20.3%)
triplet-SVM	3881	417 (10.7%)

Table 1: Sensitivity and selectivity results for raw predictions

3.3 Results on chr1 *Arabidopsis thaliana,* comparison with miRNAFold

We have also run the algorithm on the raw genomic sequence of the chromosome 1 from *Arabidopsis thaliana.* This chromosome, with a length of 35 Mb, contains 88 pre-miRNAs producing 102 mature miRNAs according to miRBase annotations. Of course, our algorithm is not intended to be used with such a large sequence without deep sequencing data. It is expected to produce a huge number of false positive predictions. The goal of this experiment is precisely to quantify this number of false positive predictions, and to see if we are still able to extract some signal.

From this dataset, we produced a total of 77,584 secondary structures. By way of comparison, the miRNAFold program [10] was launched on the same data, with the following parameters: sliding window size 400, minimum hairpin size 70, species parameters *A. thaliana*. miRNAFold is an ab initio software that searches for pre-miRNA structures in genomic sequences without any given additional information. miRNAfold predicts more than 200,000 pre-miRNAs, three times more than our algorithm.

If we look at the MFEI level (see below), 9,314 out of our 77,584 predictions have a MFEI smaller than the threshold -0.8. This subset of predictions contains 89.8% of all pre-miRNAs annotated in miRBase for chromosome 1.

4 Thermodynamic stability of the pre-miRNA and MFEI thresholds

Several publications establish that plant pre-miRNAs have distinctive structural folding characteristics compared to pseudo-hairpins (e.g. [11]). The first folding measures we use are the MFE, the MFEI and the AMFE [12].

- *MFE* (*minimal free energy*) denotes the negative folding free energy of a secondary structure. It is computed with the Matthews-Turner nearest neighbour model implemented in RNAeval [13]. This model considers the minimum energy values obtained by complementary base pairs decreased by the stacking energy of successive base pairs or increased by the destabilising energy associated with non-complementary bases.

- *AMFE* is an *adjusted MFE*. It is calculated by the equation:

AMFE = MFE / sequence length x 100.

- *MFEI* is the *minimal folding energy index*. It is calculated by the equation:

MFEI = [MFE / sequence length x 100] / GC% = AMFE/GC%

Among all these measures, the MFE, AMFE and MFEI are interdependent by nature. We chose the MFEI as major indicator.

Mirkwood uses the MFEI threshold twice.

- It has an option to keep only precursors whose MFEI is smaller than -0.6.
- Moreover, we add one star in our score system when the MFEI of the precursor is strictly smaller than -0.8.

We can take a deeper look at the MFEI distribution for the hairpin found with the positive and negative datasets of the previous section. Results are available in Table 2. They show a clear separation between the predictions coming from positive and negative samples. The optional threshold -0.6 allows to discard more than 30% of false positive predictions, while eliminating less than 0.5% sequences in the high confidence miRBase dataset. As for the -0.8 threshold, it is reached by more than 90% sequences in the positive datasets, whereas less than 5% of sequences in negative datasets reach it.

	MFEI ≥ -0.6	-0.6 > MFEI ≥ -0.8	MFEI < -0.8
Plant mirbase (5269 hairpins)	1.5%	8%	90.5%
High confidence plant miRBase (554 hairpins)	0.5%	5.8%	93.7%
plantmirRNApred (418 hairpins)	38.8%	56.2%	5%
triplet-SVM (417 hairpins)	30.2%	65%	4.8%

Table 2 : MFEI distribution

Another measure which distinguishes pre-miRNA precursors from pseudo-hairpins is the stability of the real precursor when compared to their randomised counterparts. This idea was initially introduced in randfold [11] and is used in Mirdeep-P [14].

Randfold computes the probability that, for a given RNA sequence, the MFE of the secondary structure is different from a distribution of MFE with Monte Carlo and randomisation tests. The downside of this approach is that it is time-consuming. For each candidate, it requires to generate hundreds of randomised sequences and to fold them. We have designed a custom program that concentrates on sequences with significant MFE and stops the calculation as soon the target probability is above a given threshold (set to 0.1). It uses RNAfold [13] to compute the MFE, and the Altschul-Erikson algorithm to generate randomised sequences with dinucleotide preservation.

In Figure 3, we compare the behaviour of the MFEI and of the P-value for dinucleotide shuffled sequences on all plant miRBase sequences. We observe that low values of MFEI (<-0.8) are systematically associated to low values of P-value. In this perspective, the functionality *Shuffle* is an optional criteria of miRkwood.

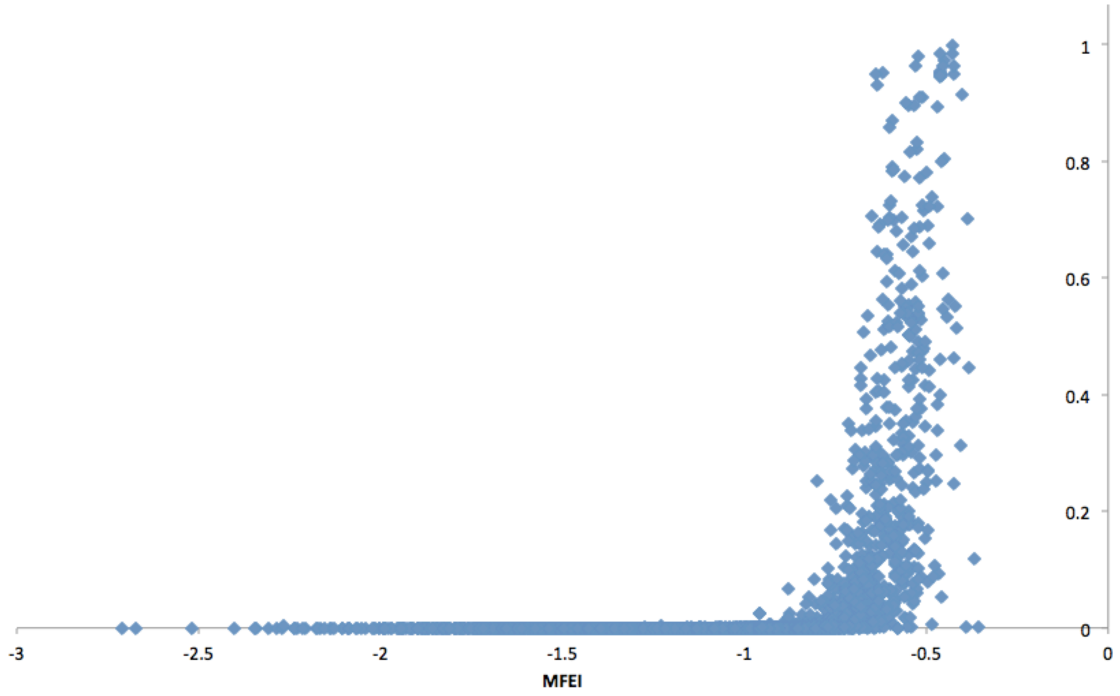

Figure 3: Relation between the MFEI and the P-value calculated on shuffled sequences for the miRBase precursor sequences.

5 Evolutionary conserved mature miRNAs

Several miRNA families are conserved between multiple plant species [15]. This observation is extensively used by microHarvester [16], for example. Such tools usually used BlastN to search for similar sequences in miRBase. Here we have chosen to use Piccolo [17], that is an exact implementation of the sequence alignment problem. It is more sensitive than BlastN, while keeping the computational time low. Moreover, it guarantees that the comparison involves the full length mature miRNA.

We compare the precursor sequence to the set of miRNA sequences of miRBase V21 such as available in the file 'mature.fa' restricted to the clade *Viridiplantae*, and we select all alignments with at most three errors (mismatch, deletion or insertion) that occur in one of the two arms of the stem-loop. Three errors with the file miRBase mature.fa corresponds to an empirical estimated P-value of $3E^{-2}$ for each pre-miRNA. Alignments with 2 errors or less have an estimated P-value of $4E^{-3}$. Moreover, we check whether the alignment overlap with the miRNA.

References

1. Smith TF, Waterman MS. Identification of common molecular subsequences. J Mol Biol. 1981;147:195–7.

2. Kozomara A, Griffiths-Jones S. miRBase: integrating microRNA annotation and deep-sequencing data. Nucleic Acids Res. 2011;39 Database issue:D152–7.

3. Hofacker IL, Fontana W, Stadler PF, Bonhoeffer LS, Tacker M, Schuster P. Fast folding and comparison of RNA secondary structures. Monatshefte Für Chem Chem Mon. 1994;125:167–88.

4. Hofacker IL, Priwitzer B, Stadler PF. Prediction of locally stable RNA secondary structures for genome-wide surveys. Bioinforma Oxf Engl. 2004;20:186–90.

5. Xuan P, Guo M, Liu X, Huang Y, Li W, Huang Y. PlantMiRNAPred: efficient classification of real and pseudo plant pre-miRNAs. Bioinforma Oxf Engl. 2011;27:1368–76.

6. Gudyś A, Szcześniak MW, Sikora M, Makałowska I. HuntMi: an efficient and taxon-specific approach in pre-miRNA identification. BMC Bioinformatics. 2013;14:83.

7. Khalifa W, Yousef M, Saçar Demirci MD, Allmer J. The impact of feature selection on one and two-class classification performance for plant microRNAs. PeerJ. 2016;4:e2135.

8. Xue C, Li F, He T, Liu G-P, Li Y, Zhang X. Classification of real and pseudo microRNA precursors using local structure-sequence features and support vector machine. BMC Bioinformatics. 2005;6:310.

9. Ng KLS, Mishra SK. De novo SVM classification of precursor microRNAs from genomic pseudo hairpins using global and intrinsic folding measures. Bioinforma Oxf Engl. 2007;23:1321–30.

10. Tav C, Tempel S, Poligny L, Tahi F. miRNAFold: a web server for fast miRNA precursor prediction in genomes. Nucleic Acids Res. 2016;44:W181-184.

11. Bonnet E, Wuyts J, Rouzé P, Van de Peer Y. Evidence that microRNA precursors, unlike other non-coding RNAs, have lower folding free energies than random sequences. Bioinforma Oxf Engl. 2004;20:2911–7.

12. Zhang B, Pan X, Cox S, Cobb G, Anderson T. Evidence that miRNAs are different from other RNAs. Cell Mol Life Sci CMLS. 2006;63:246–254.

13. Lorenz R, Bernhart SH, Höner zu Siederdissen C, Tafer H, Flamm C, Stadler PF, et al. ViennaRNA Package 2.0. Algorithms Mol Biol. 2011;6:26.

14. Yang X, Li L. miRDeep-P: a computational tool for analyzing the microRNA transcriptome in plants. Bioinforma Oxf Engl. 2011;27:2614–5.

15. Cuperus JT, Fahlgren N, Carrington JC. Evolution and functional diversification of MIRNA genes. Plant Cell. 2011;23:431–42.

16. Dezulian T, Remmert M, Palatnik JF, Weigel D, Huson DH. Identification of plant microRNA homologs. Bioinformatics. 2006;22:359–60.

17. Vroland C, Salson M, Bini S, Touzet H. Approximate search of short patterns with high error rates using the 01*0 lossless seeds. J Discrete Algorithms. 2016;37:3–16.
